# Table-Balancing Cooperative Robot Based on Deep Reinforcement Learning

**DOI:** 10.3390/s23115235

**Published:** 2023-05-31

**Authors:** Yewon Kim, Dae-Won Kim, Bo-Yeong Kang

**Affiliations:** 1 Department of Artificial Intelligence, Kyungpook National University, Daegu 41566, Republic of Korea; yewonkim.knu@gmail.com; 2 School of Computer Science and Engineering, Chung-Ang University, 84 Heukseok-Ro, Seoul 06974, Republic of Korea; dwkim@cau.ac.kr; 3 Department of Robot and Smart System Engineering, Kyungpook National University, Daegu 41566, Republic of Korea

**Keywords:** reinforcement learning, deep Q-network, cooperative robot, human–robot interaction

## Abstract

Reinforcement learning is one of the artificial intelligence methods that enable robots to judge and operate situations on their own by learning to perform tasks. Previous reinforcement learning research has mainly focused on tasks performed by individual robots; however, everyday tasks, such as balancing tables, often require cooperation between two individuals to avoid injury when moving. In this research, we propose a deep reinforcement learning-based technique for robots to perform a table-balancing task in cooperation with a human. The cooperative robot proposed in this paper recognizes human behavior to balance the table. This recognition is achieved by utilizing the robot’s camera to take an image of the state of the table, then the table-balance action is performed afterward. Deep Q-network (DQN) is a deep reinforcement learning technology applied to cooperative robots. As a result of learning table balancing, on average, the cooperative robot showed a 90% optimal policy convergence rate in 20 runs of training with optimal hyperparameters applied to DQN-based techniques. In the H/W experiment, the trained DQN-based robot achieved an operation precision of 90%, thus verifying its excellent performance.

## 1. Introduction

With recent technological advancements, various machines and robots with artificial intelligence (AI) have emerged to work on behalf of people, ranging from washing machines to robot cleaners. Reinforcement learning (RL) [1,2,3,4,5], an artificial intelligence technique, is commonly applied in robot action learning. Several prior studies on RL have been conducted to develop behavior-imitation robots [6,7] and robot navigation [8,9]. As AI robots undertake human tasks, individuals have more time for leisure and self-development; as a result, many people require robots to work instead of humans.

Daily human tasks are divided into those that can be completed alone and those requiring cooperation between two or more people, such as moving an object. In most previous studies using RL, robots perform tasks on their own on behalf of humans [8,9,10,11,12,13,14,15,16,17]. Various human–robot interaction (HRI) technologies [18,19,20,21,22,23,24,25,26,27] are being studied, but most of them focus on research for robots to engage in conversation with humans [18,19,20,21,22,23] or utilize human feedback for robot learning [26,27] rather than collaborating with humans to perform tasks. In the industry, cobots (collaborative robots) are robots that cooperate with humans without separating barriers, but most of them are fixed in specific places. For such a robot to move objects on behalf of humans, cooperation is required to balance objects with opponents holding objects. For instance, when moving objects such as tables and televisions, it is necessary to balance objects parallel to the ground for free movement and avoid damaging them. When a robot moves objects on behalf of humans, cooperation is required to balance objects with opponents holding objects. Cooperative object-balancing methods were proposed in [28,29]. However, they are only limited to high-performance devices and semi-automatic systems requiring human intervention.

Therefore, in this paper, we propose a deep-reinforcement-learning-based cooperative robot technique for automatic table balancing using a general-purpose camera on a humanoid robot. The proposed method’s operating principles are as follows. While a human and a robot are holding a table, the robot recognizes the balance of the table that varies depending on the human’s movements and adjusts the balance through appropriate actions. The robot recognizes human actions via the camera and performs a balancing operation appropriate for the state based on the policy that the robot learned.

In this paper, we used a humanoid robot, NAO V3.3 [30] (head V4.0, other parts V3.3), to cooperate with a human. The reason for using a humanoid robot is that, for the purpose of moving objects with humans, the robot needs to be able to hold objects with its hands and move with its feet. The NAO used in this study is equipped with two MT9M114 cameras on its head, with the upper camera facing forward and the lower camera facing downward. In this experiment, we utilized the lower camera, and detailed explanations for why we used the lower camera are provided in Section 3. The NAO V3.3’s joints are controlled by two types of motors, RE-Max 24 and RE-Max 17, with RE-Max 24 used in the legs and RE-Max 17 used elsewhere.

This paper is organized as follows. Section 2 explores RL technology and previous studies using reinforcement learning on robots, and Section 3 describes the proposed method. In Section 4, we discuss the results of the experiments with the proposed technique. Finally, Section 5 concludes this study and suggests future research directions.

## 2. Previous Work

RL does not require detailed expert supervision for all actions since it learns tasks through rewards based on the agent’s trial and error. As a result, an RL agent sometimes learns higher skills than humans. An example of this is how a deep Q-network (DQN)-based agent achieved a high score in the Atari Breakout game [1] by learning to use the ball to dig a tunnel through the center and sides of the brick, such that the ball repeatedly bounces between the wall and the brick. With these out-of-the-box characteristics, various problems have adopted RL to solve or train intelligent agents. RL has been studied in a variety of ways, beginning with deep Q-network (DQN) [1], which combines neural networks into Q-learning [2]. Since DQN, reinforcement learning techniques have been studied to improve training stability, such as trust region policy optimization (TRPO) [3] and proximal policy optimization (PPO) [4], which are updated by approximating the region of trust, and deep deterministic policy gradient (DDPG) [5] for continuous behavior of the agent.

AlphaGo [10] is a well-known application of applying RL to a game, competing against world-famous Go players. AlphaGo (David Silver et al. [10]) improved its performance by combining deep reinforcement learning with tree search, which is mainly used for Go AI. As a result, AlphaGo won a number of victories against famous Go players. Like AlphaGo, which played the game on its own, there are many studies in which robots learned natural actions through RL, and behavior-imitation studies include human behavior imitation of humanoid robots [6] and animal behavior imitation of animal robots [7]. By comparing pre-recorded human gaits with the generated behavior, Josh Merel et al. [6] achieved a more natural human gait for humanoid robots via RL imitation learning. X. B. Peng et al. [7] researched animal robot behavior to learn behavior driving suitable for terrain change using animal behavior data and RL.

RL has been used for navigation robot research [8,9]. In the RoboCup Standard Platform League (SPL), Kenzo Lobos-Tsunekawa et al. [8] implemented a mapless visual navigation system in which robots used RL-generated paths without map information. Shuhuan Wen et al. [9] investigated a navigation robot that used Q-learning and an extended Kalman filter for simple localization and mapping (EKF-SLAM). In this previous research, RL-based path finding for autonomous robots was developed.

Robots used RL to perform tasks such as bowling [11], standing up [13], balancing and opening doors [14], catching and moving objects [15,16], and using trash cans [17]. Debnath and Nassour [11] improved the performance of bowling skills for a NAO robot with RL by exploiting successful actions and exploring unsuccessful actions. M. Daniel [13] investigated pose transition from lying to standing for an NAO robot using RL. Freek Stulp et al. [14] applied RL to robot learning of two tasks: opening the door and balancing the body based on the right arm operation. By recognizing 1100 different item kinds with cameras, Sergey Levine et al. [15] developed object grip and transport behavior that was optimized for a 7 DOF manipulator with RL. Robots learn more quickly when humans provide guidelines, as demonstrated by Suay and Chernva [16]’s use of RL to transfer objects to the left or right cup. Chang Wang et al. [17] used color image segmentation to indicate the degree of trash can opening while learning how to use the trash can from camera images with RL. The advantage of previous studies is that robots can operate autonomously indoors; however, those studies are unsuitable for cooperative missions because one robot is satisfied with proper actions without optimal action exploration, another requires landmarks to check its current position, and robots work alone in stable situations without any changes from humans or partners.

Additionally, researchers have proposed various HRI techniques [18,19,20,21,22,23,24,25,26,27]. These preexisting HRI techniques span from empirical social rules [18,19,20] to an end-to-end learning framework [21,22,23,26,27]. For the empirical social rules, refs. [18,19] proposed a communication-initiation method by classifying whether a human has an intention to interact with a robot. Bergstrom et al. [20] also proposed a classification of the movement and interests of visitors around the robot using a laser range finder. However, these empirical social rules require specific scenarios and sophisticated sensors. Refs. [21,22] generated robot–human greetings using imitation learning for the end-to-end learning framework. N. T. V. Tuyen et al. [21] trained the robot to express body language, and M. Doering et al. [22] trained a robot to recommend travel packages as a travel agency staff member. However, the above end-to-end framework studies have limitations in that the generated greetings are limited to fewer than ten and suffer from unnaturally formed conversations. To activate natural conversation timing, Yang Xue et al. [23] have developed a technology that allows the robot to autonomously perceive and converse or guide a human without a start command using vision. However, the process of creating datasets for learning includes costly expert timing for when to talk. For human activity recognition (HAR), several methods are proposed [24,25]. Beril Yalçinkaya et al. [24] enhanced the predictability of human activity sequences by combining long short-term memory (LSTM) and fuzzy logic. Imran Ullah Khan et al. [25] developed a hybrid model by incorporating a convolutional neural network (CNN) and LSTM for activity recognition, where the CNN is used for spatial feature extraction and the LSTM network is utilized for learning temporal information. Other learning-based approaches include that of Huang and Mutlu [26], which used dynamic Bayesian networks (DBNs) to model how robots adjust to a person’s voice, gaze, and gestures in narration during human–robot learning. Ta- Chung Chi et al. [27] applied a technique that allows the navigating agent to ask for human feedback about uncertain prediction results during reinforcement learning training. Nonetheless, human feedback for learning requires continuous human intervention, leading to high feedback costs. Wen Qi et al. [31] recognized hand gestures using sensor fusion with LSTM-RNN. In [32], data gathered from the chest band were saved on an iPhone 6 and then transmitted to a computer via wireless networks. However, this required not only a robot and a device but also additional installation of sensors such as a depth vision sensor, EEG, GPS, etc.

The adoption of cobots in the industry for tasks such as picking, placing, and assembling parts is on the rise. Thus, various studies are being conducted for its betterment. Hang Su et al. [33] developed a human-activity-aware adaptive shared control solution for human–robot interaction in surgical operations. Dorothea Schwung et al. [34] considered the problem of heterogeneous cooperative multi-robot control using an RL-based algorithm. Although these techniques are powerful enough to allow humans to control or hold objects stably, these are possible because the robots are immobile on fixed floors. Control of mobile robots, such as humanoids, requires more complex manipulation. As [34] mentioned, multi-agent systems have developed in variable ways. Yara Rizk et al. [35] are especially focused on heterogeneous multi-agent systems (MAS). J. Krüger et al. [36] proposed a framework to control stable and robust bimanual-manipulation tasks for a dual-arm robot. Ruihua Han et al. [37] implemented a platform for multi-robot navigation in dynamic environments using deep reinforcement learning to find the optimal path for robots. Most proposed multi-robot systems recognize human motion to prevent human damage or to work with humans. However, these robots communicate only with robots, including systems to control multi-agents without human interaction.

Furthermore, researchers have investigated human–robot cooperation. In [28,29], the human–robot cooperative task of table balancing was performed. The table-balancing technology of the robot is composed of two controllers: a human-motion-prediction controller and a robot motion controller for table balancing. Both controllers used table coordinate information to output the results, and the coordinate information of the table was obtained with the Vicon MX motion capture system [38], which included optical marker labeling and multiple high-resolution cameras. However, the limitations of this study, which collected table coordinate information, are that several high-performance cameras are demanded in the Vicon MX motion capture system, and semi-automatic systems requiring human intervention for marker labeling are also required.

To overcome these limitations, this paper utilizes vision technologies and a universal camera mounted on robots to perform balancing tasks automatically without using high-performance devices. In addition, the robot learns to interact with a human to perform a table-balancing task without high-cost human feedback. We propose a table-balancing cooperative robot technology based on RL. The proposed robot uses deep reinforcement learning to recognize the action state of the human who moved the table as an image, then performs the action and learns the optimal table-balance action. Furthermore, it is possible to cooperate with humans by recognizing human actions in real time and performing table-balancing actions.

## 3. Proposed Table-Balancing Cooperative Robot Based on Reinforcement Learning

**Figure 1:**
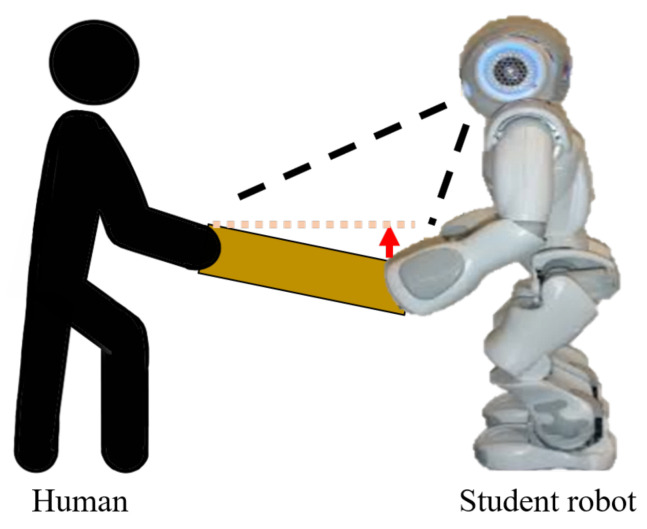
Table-balancing task.

**Figure 2:**
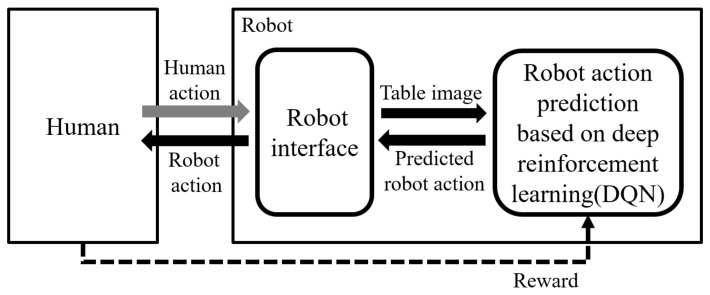
Proposed reinforcement learning framework between human and robot for table balancing.

The table-balancing task, as depicted in Figure 1, involves the robot cooperatively balancing the table by recognizing the unbalanced table when both the human and the robot are holding the table. To successfully perform cooperative balancing tasks, this study proposes a balancing method based on reinforcement learning, and the overall workflow is shown in Figure 2. First, when a human performs an action, the robot interface captures a table image and sends it to component of the robot action prediction, which is based on deep reinforcement learning. The robot action-prediction component recognizes the current human action with the table image and predicts the robot action to be appropriate for the current state. The robot drives the predicted robot action, and the environment sends a reward to the robot action-prediction component based on the new state changed by the robot action. Finally, the reward-based robot action-prediction component is updated. That is, the robot is rewarded for the good or bad robot action predicted and learns to output the optimal action in each state. The robot action-prediction component is achieved using DQN, a deep reinforcement learning algorithm.

**Figure 3:**
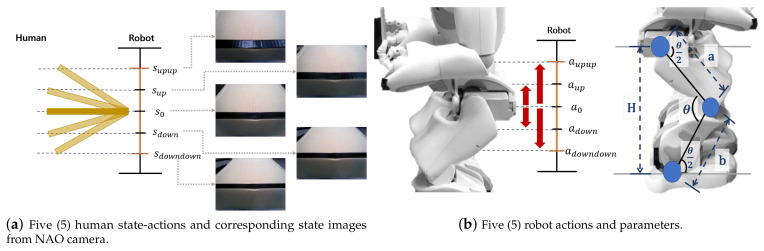
Human state-action images and robot actions.

The role of the robot interface (Figure 2) in this technology is literally to connect the outside and inside of the robot. The details of its working are shown in Figure 3. Through the robot interface, the robot captures table images (Figure 3a) and calculates and drives robot motions (Figure 3b).

Given the table image from the robot interface, the principle of recognizing the human action is shown in Figure 3a. When a human and a robot hold a table, the robot can see the top of the table and the human action changes the shape of the table in the robot’s view. In this paper, human actions are classified into five categories based on the degree of raising and lowering the table: up a lot ($s_{upup}$), up ($s_{up}$), keep ($s_{0}$), down ($s_{down}$), and down a lot ($s_{downdown}$). In the robot’s eyes, for example, when a human raises a table ($s_{upup}$, $s_{up}$), the area of the table increases in comparison to when the human lowers the table ($s_{down},s_{downdown}$). As a consequence, the robot recognizes the human action. In this case, the robot’s sight is like classifying table shapes from table images.

Figure 3b shows the robot behavior and parameters for its calculation. As shown in the image on the right-hand side of Figure 3b, there are five robot actions, $a_{downdown},a_{down},a_{0},a_{up}$, and $a_{upup}$, which are divided according to the degree to which the robot’s hip goes up or down, as in the state. When manipulating a robot arm, the complexity of the problem increases as it requires modeling various balance states corresponding to different robot arm positions. To solve this problem, a robot was constructed that restricts its arm movements and achieves table balancing solely through the use of its legs. In addition, we only consider the slope, excluding table movement, and assume that the slope of the surface held by the robot or person with both arms is the same. As shown in Figure 3b, the hip height (*H*) is calculated using the current leg angle to classify the behavior according to the robot’s hip position. The height to be operated is added, and the new robot leg angle is calculated and driven. The height of the hip is calculated using the cosine, which is Equation (1).

(1) $$H=\sqrt{a^{2}+b^{2}-2abcos\theta_{knee}}$$

where a,b are the length of the NAO robot’s thigh and calf links, 100 mm and 102.9 mm, respectively. $\theta_{knee}$ is the robot knee angle, the current knee angle obtained from the robot’s built-in system. Prior to calculating the new leg joint angle according to the robot drive height, we empirically experienced that the motion angle of the hip, knee, and ankle must be balanced for the robot to drive reliably. As shown in Figure 3b, the motion angles of the hip and ankle ($\theta_{hip},\theta_{ankle}$) should only be moved half of the motion angle of the knee ($\theta_{knee}$) so that the robot operates stably without falling over. If the angle is maintained as above, the two gray lines around the hip and ankle in Figure 3b are to be parallel with the ground. Then, the posture balance of the robot is stable. The computation of the knee angle ($\theta_{knee}$) for robot motion utilizes the inverse cosine function, which is Equation (2). In addition, the angles of the hip and ankle ($\theta_{hip},\theta_{ankle}$) are expressed in Equation (3).

(2) $$\theta_{knee}=\arccos\frac{a^{2}+b^{2}-{\left(H+h\right)}^{2}}{2ab}$$

(3) $$\theta_{hip}=\theta_{ankle}=\frac{\theta_{knee}}{2}$$

**Figure 4:**
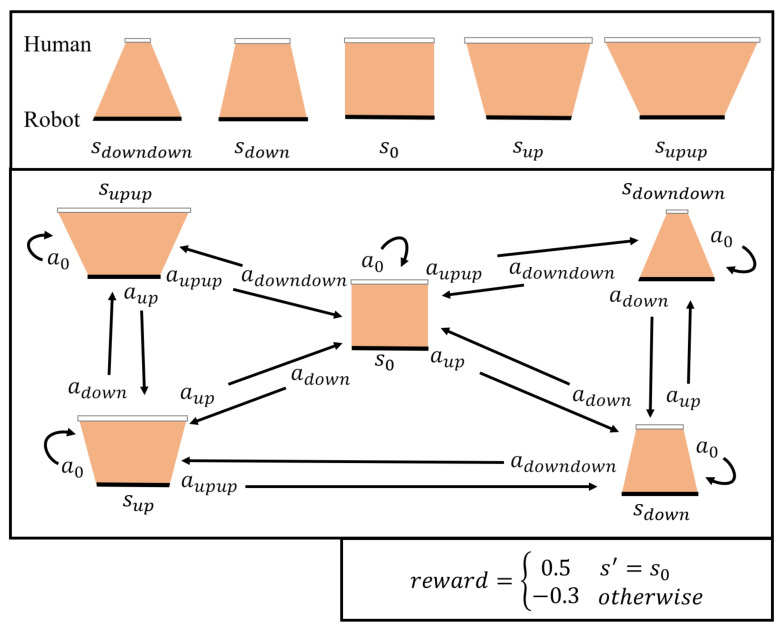
Five (5) human action states from table image and reward model of the table-balancing task.

Figure 4 depicts a reward model for robot learning based on human action, with each state showing the probable robot actions to transit to the next state. If the table is balanced, a positive reward (0.5) is given; otherwise, a negative reward (−0.3) is given.

As earlier mentioned, DQN is a deep reinforcement learning technique. Fundamentally, it combines artificial neural networks with Q-learning to approximate the action-value function (Q-function); however, by adding a convolutional neural network (CNN) its prediction of image-based states for action learning is more refined.

**Figure 5:**
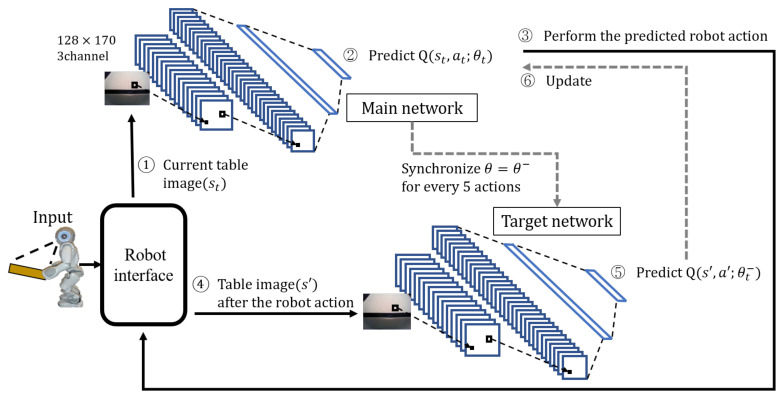
DQN learning process for table balancing.

Figure 5 depicts the learning process of DQN’s robot action prediction for the proposed table-balancing cooperative robot. First, the robot interface photographs the current table image and sends it to the DQN’s main network (①). The main network predicts the Q-function for the robot action using the table image (②) and determines the robot action using Equation (4)’s ε–greedy policy.

(4) $$\pi \left(s\right)=\left\{\begin{array}{cc} {\mathrm{argmax}}_{a\in A}Q\left(s,a\right) & n>\varepsilon \\ \mathrm{random}\;\mathrm{action} & n\leq \varepsilon \end{array}\right.$$

In Equation (4), π represents a policy, *s* represents a state, *a* represents a robot action, and *A* represents a set of all robot actions; in this paper, the five robot actions are $a_{downdown},a_{down},a_{0},a_{up},a_{upup}$. argmax${}_{a\in A}Q\left(s,a\right)$ returns the robot action (*a*) with the highest Q-function value in that state (*s*). The probability of random action is denoted by ε. *n* is a random variable. When several robot actions equally have the maximum Q value, one of them is chosen at random (③).

In RL, if the agent learns only with exploitation, it could only learn the successful action in the early stages, not the optimal policy. To solve this problem, we explore other actions at random in Equation (4) to learn the optimal policy.

The same network structure is adopted for both the main and target networks, with the details as follows:

- 16-channel convolution layer, 8 × 8 filter, stride = 4
- 32-channel convolution layer, 4 × 4 filter, stride = 2
- 256-unit fully-connected layer
- 5-unit fully-connected layer

The five actions predicted by the Q-function are represented by the last five-neuron fully connected layer in both the main and target networks. After performing the robot action predicted by the robot (③), the table image is retaken from the robot interface (④) to predict the Q-function of the new state after the robot action in the target network (⑤) and update the main network using it (⑥). The following is the main network update: the main network’s loss function uses the mean squared error (MSE), as shown in Equations (5) and (6).

(5) $$J\left(\theta_{t}\right)=\frac{1}{2}{\left(y_{t}-Q\left(s_{t},a_{t};\theta_{t}\right)\right)}^{2}$$

(6) $$y_{t}=\left\{\begin{array}{cc} r_{t} & \mathrm{if}\;\mathrm{episode}\;\mathrm{done}\;\mathrm{at}\;t+1\\ r_{t}+\gamma Q\left(s^{\prime },a^{\prime };\theta_{t}^{-}\right) & \mathrm{otherwise} \end{array}\right.$$

*J* represents a loss function, *y* represents a target function, and *t* of $s_{t},a_{t}$ represents an episode. $s^{\prime }$ is the next state that has driven $a_{t}$ at $s_{t}$. $Q(s^{\prime },a^{\prime };\theta_{t})$ is the action-value function as determined by the main network. The target network predicts the future action-value function $Q(s^{\prime },a^{\prime };\theta_{t}^{-})$ of the target function *y*. The weights of the main and target networks are denoted by θ and $\theta^{-}$, respectively. Every five episodes (*t*), the target network is updated in synchrony with the main network. $r_{t}$ is a reward for the robot action, and γ is a discount factor that reduces the effect of the following states on the Q-value, with a value between 0 and 1.

In Equation (6), the future rewards may be considered high if the influence of the following states ($Q(s^{\prime },a^{\prime };\theta_{t}^{-})$) is too large. If the influence is too small, the agent may not be able to learn the optimal action because it focuses only on the present rewards. Therefore, it is important to determine the appropriate discard factor.

Drastic updates can make the optimal policy convergence slow or fail to converge; as such, Equation (7) is a gradient clipping that adjusts the amount of updates to prevent these drastic updates.

(7) $${\hat{g}}_{t}=\left\{\begin{array}{cc} \frac{\mathrm{threshold}}{|{▽}_{\theta_{t}}J\left(\theta_{t}\right)|}{▽}_{\theta_{t}}J\left(\theta_{t}\right) & \mathrm{if}\;|{▽}_{\theta_{t}}J\left(\theta_{t}\right)|\geq \mathrm{threshold}\\ {▽}_{\theta_{t}}J\left(\theta_{t}\right) & \mathrm{otherwise} \end{array}\right.$$

$\hat{g}$ is a loss function for the clipped gradient, and the threshold is set to be the maximum loss limit. Finally, we update the main network using a stochastic gradient descent (SGD) in Equation (8).

(8) $$\theta_{t+1}=\theta_{t}-\alpha {\hat{g}}_{t}$$

α is the learning rate determining the weight update degree. If α is too high, it may not converge; if it is too low, it slows down the training time.

By repeating this process, DQN learns robot action prediction for table balancing under the constant effect of the model (Figure 4). As a result, the robot has a consistent final policy that predicts the appropriate robot action in each state.

## 4. Experimental Results

This section describes the experimental results used to validate the proposed table-balancing cooperative robot based on deep reinforcement learning. This experiment is described in the following order: constructing a dataset for predicting the robot action, determining the optimal policy convergence performance based on DQN parameter changes, and demonstrating the robot H/W performance. Softbank’s NAO robot [30] was used in this paper, and the table is a rectangular table that is 31 cm wide, 23 cm long, and 6 cm high, making it easy for the NAO robot to hold.

### 4.1. Dataset Construction

The dataset [39] created for robot action prediction is contains table state images tagged with five human action states, as shown in Figure 3a. We generated a total of 7174 sheets of table images for training the robot action-prediction component, as well as a dataset, by photographing table changes based on human actions with a robot camera and converting them to 320 × 240 images.

In taking these ground-truth images for each state ($s_{upup},s_{up},s_{0},s_{down},s_{downdown}$), the table was set at 6°, 1° to 6°, 1° to −1°, −1° to −6°, and −6°, respectively.

**Figure 6:**
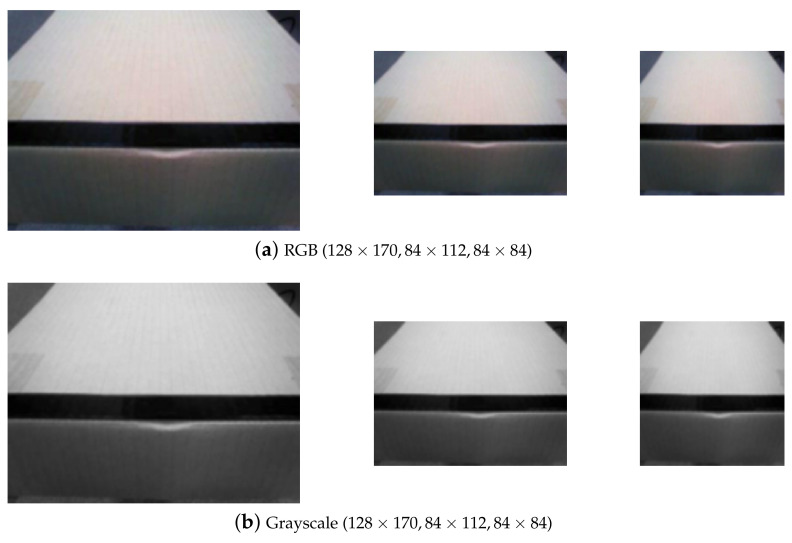
Preprocessing images (RGB, grayscale).

An experiment was conducted to find an image optimized for robot learning, and the image used in the comparative experiment is shown in Figure 6, with the RGB image (original) and grayscale images adjusted to 128 × 170, 84 × 112, and 84 × 84.

If the image size is large, the computational cost increases. If it is small, the input information may not be sufficient, so it is necessary to determine the appropriate size.

Table 1 displays the results of experiments conducted on image size and color to be used for DQN training. In this image preprocessing experiment, the learning rate α was 0.005, the discount factor γ was 0.9, the random action probability ε was 10%, and the episode was repeated 50,000 times. The optimal policy convergence rate (OPCR) was measured by repeating the process 30 times for each image in Figure 6. In Table 1, the OPCR was observed to be 90 percent when using 128 × 170 RGB images, demonstrating the best performance when compared to other preprocessing images, and 84 percent convergence when using 84 × 112 grayscale images. According to the experimental results, the DQN training image was a 128 × 170 RGB image.

Parameter-search experiments for the optimal learning rate α and discount factor γ were conducted for the proposed method, that is, the greedy policy, the 10–greedy policy, and the 20–greedy policy. Training DQN with this proposed method means the greedy policy will always select the actions with the highest Q-value, while the 10–greedy policy and the 20–greedy policy select random actions with 10% and 20% probability, respectively.

The learning rate α and discount factor γ in each policy were changed from 0.001 to 0.009 and from 0.1 to 0.9, respectively, and all experiments were repeated 20 times, 50,000 episodes per parameter change. Hyperparameter tuning is important because it causes the best training performance and training time.

**Figure 7:**
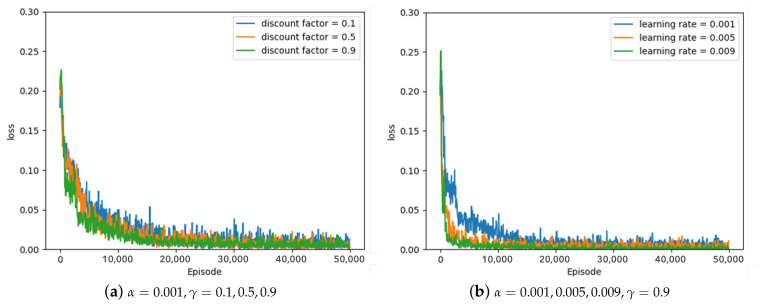
Greedy policy, loss graph of DQN with parameter changes (α = 0.001∼0.009, γ = 0.1∼0.9).

**Figure 8:**
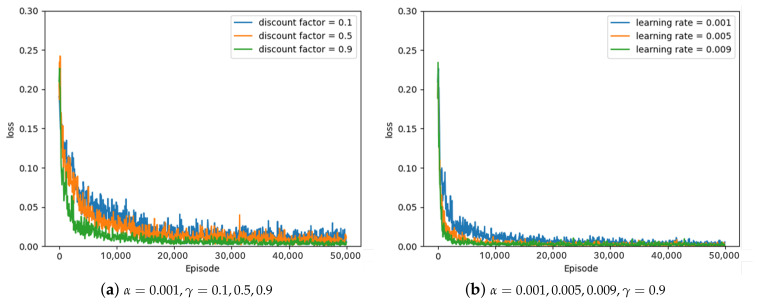
10–greedy policy, loss graph of DQN with parameter changes (α = 0.001∼0.009, γ = 0.1∼0.9).

**Figure 9:**
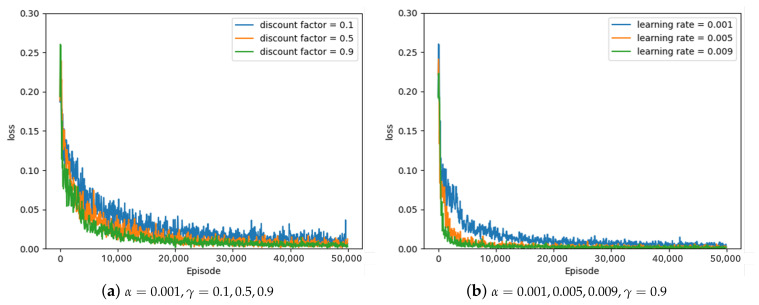
20–greedy policy, loss graph of DQN with parameter changes (α = 0.001∼0.009, γ = 0.1∼0.9).

Some of the experimental results from the optimal parameter experiment are depicted in Figure 7, Figure 8 and Figure 9. Figure 7, Figure 8 and Figure 9 represent the average loss (Equation (5)) per 100 episodes. Figure 7 depicts the results of the greedy policy, Figure 8 depicts the results of the 10–greedy policy, and Figure 9 depicts the results of the 20–greedy policy. First of all, Figure 7a demonstrates a loss graph with a learning rate of 0.001 and a discount factor of 0.1 (blue line), 0.5 (orange line), and 0.9 (green line). The decreasing loss indicates that the policy is converging through learning, and when the loss converges, it is analyzed to determine the policy. Figure 7a shows that when the discount factor is 0.9, the loss convergence is the fastest, and the loss convergence appears rapidly as the discount factor increases. The target $y_{t}$ in Equation (5) depends on the discount factor, which affects the amount of the next-state information in the current state. As the discount factor increases and amount of the next-state information decreases, the convergence rate improves. As illustrated in Figure 7b, the convergence rate is fastest when the learning rate is 0.009, and the convergence rate increases as the amount of updates increases in proportion to the learning rate. According to the greedy policy parameters, the same pattern can be seen in Figure 8 and Figure 9, showing the 10–greedy policy and 20–greedy policy, respectively.

### 4.2. Optimal Policy Convergence Performance with DQN Parameter Variation

**Figure 10:**
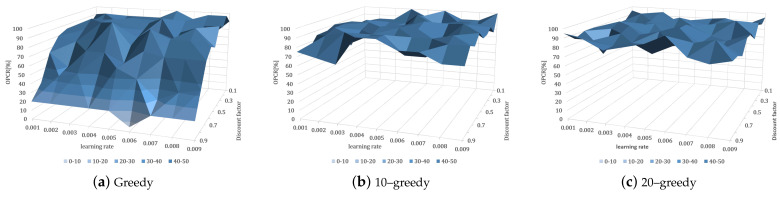
Optimal policy convergence rate (OPCR) [%] of DQN according to various hyperparameters and policies.

Figure 10 depicts the experimental results for examining the convergence performance to the optimal policy and expressing the OPCR in a 3D graph after 20 repetitions of 50,000 episodes in each policy and parameter. The optimal policy-learning ratio denotes the rate at which the policy with the parameter condition converges to the optimal policy after 20 instances of learning under the same conditions, and the optimal policy denotes the robot actions that successfully reach the table-balancing state ($s_{0}$) in each table state (*s*) in Figure 4.

Figure 10a depicts the greedy policy, Figure 10b depicts the 10–greedy policy, and Figure 10c depicts the 20–greedy policy. In Figure 10a, with the greedy policy, our model shows a higher OPCR in lower discount factor settings. On the other hand, in Figure 10b,c, with the 10–greedy policy and 20–greedy policy, respectively, the model achieved a high OPCR in most learning rates and discount factors. Equation (6) shows that when using the greedy policy, the higher the discount factor, the greater the impact on the future state, so that other actions can be updated to have a higher value than the optimal policy. This appears to be due to a lack of exploration, with the 10–greedy policy and 20–greedy policy exploring 10% and 20% of the total action selection, respectively, allowing convergence to be optimal without being biased toward the effects of future states. According to the above learning results, a quick convergence of policy is observed at a 0.009 and 0.9 learning rate and discount factor, respectively, thus making great parameters for the 10–greedy policy. However, Figure 10b shows that the OPCR is lower. The reason why the optimal policy-learning ratio is lower than that of the surroundings at the learning rate of 0.009 and the discount factor of 0.9 is predicted to be due to rapid updates, so it is necessary to investigate a lower learning rate. We concluded that a learning rate and discount factor of 0.005 and 0.9, respectively, in the 10–greedy policy achieved stable optimal policy learning for the robot. Specifically, because the OPCR is highest at 90%, a similar convergence rate is observed at a 0.009 learning rate (Figure 8b). The OPCR of each greedy policy is averaged in Table 2. As previously noted, the OPCR is low due to a lack of exploration, whereas the 10–greedy policy and 20–greedy policy are sufficiently explored, resulting in a high OPCR of 90%. The policy convergence is delayed as the number of explorations increases, so the degree of exploration is deemed sufficient for a 10–greedy policy.

Based on a series of experimental results, the proposed cooperative robot used the fastest and highest convergence rate to the optimal policy in table-balancing learning, a learning rate α of 0.005, a discount factor γ of 0.9, and a 10–greedy policy.

### 4.3. Prototype Robot Based on DQN

**Figure 11:**
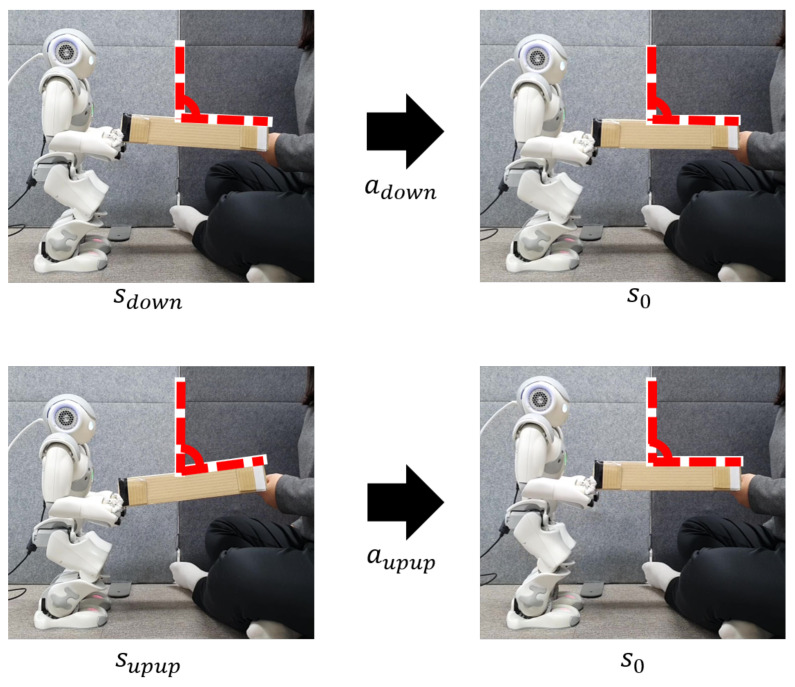
Robot demonstration based on DQN for table balancing (robot demonstration video link, https://youtu.be/ovBOF1mxf-A), accessed on 17 December 2020.

Figure 11 shows the H/W demonstration of cooperative table balancing between human and robot using the DQN-trained RL agent. Through a series of experiments, the RL agent learns under optimal conditions. The purpose of the H/W demonstration experiment is to evaluate the balancing precision of this proposed technique. When a human operates one of the operating states in Figure 3a, the robot drives the table-balancing action using Equations (2) and (3). Two examples in Figure 11 show that when a human slightly lowers a table ($s_{down}$), the robot slightly lowers the table, and when a human significantly raises the table ($s_{upup}$), the robot also drives a suitable robot action according to the DQN policy trained in the human action state. Table 3, which is the result of the numerical representation of the two examples, clearly indicates the robot’s performance and also the baseline’s performance (a cooperative robot based on Q-learning with AlexNet [39]). The measured table angle after calibration is shown in Table 3, so that the table and the ground are parallel when the table slope is 0°. For instance, when the angle is greater than 0° ($s_{up,}s_{upup}$), the table is higher in the human direction than in the robot direction, and vice versa when the angle is less than 0° ($s_{down},s_{downdown}$). When the table was unbalanced due to human action at 7.47° and −2.64°, our model was able to predict the appropriate action for the robot to balance the table ($s_{0}$) with a table angle of 0.66° and −0.30°.

In a total of 100 real-time tests, the DQN-based robot drove the optimal table-balancing action 90 times, and the operation precision for measuring the robot’s balancing performance is shown in Equation (9).

(9) $$\mathrm{Precision}=\frac{\mathrm{num}.\;\mathrm{of}\;\mathrm{balanced}\;\mathrm{action}}{\mathrm{num}.\;\mathrm{of}\;\mathrm{trial}\;\mathrm{action}}$$

The DQN-based cooperative robot’s real-time table-balancing precision calculated using Equation (9) was 90% [40], indicating an excellent performance better than 67% [39]. Furthermore, even in a number of tests where the table was not balanced, the DQN-based robot performed a robot action similar to the optimal table-balance action. This result indicates that the robot correctly analyzed the human action state. The H/W demonstration results of DQN-based cooperative robots are provided at the following link: https://youtu.be/ovBOF1mxf-A, accessed on 17 December 2020.

## 5. Conclusions

This study proposed an RL-based method that allows a human and a robot to maintain table balance collaboratively. The human behavior was recognized via the captured table image using a universal camera built into the robot. This has overcome the limitations of previous research, such as the demand for high-performance devices, human intervention in semi-automatic systems, and additional installation of sensors. To validate the performance of the proposed method, dataset construction, table image experimentation, and parameter experimentation were performed, and the proposed technique quickly converged to the optimal policy for table balancing using an RGB image input of size 128 × 170, a learning rate of 0.005, and a depreciation rate of 0.9 with a 10–greedy policy. Furthermore, the superiority of the proposed technique was demonstrated by the fact that the table-balance precision was observed to be 90% in the H/W test of the actual robot learning under the aforementioned conditions. Our proposed method contributes to the field of human–robot interaction through a human and a robot working together to balance a table simultaneously in front of each other. Future research will expand on the proposed method to broaden the range of human action states and robot actions, as well as to implement a more delicate human–robot balancing method and apply it in real life by advancing the learning method. An extended study of the customized table-balancing-robot method, voice-interactive reinforcement learning for table-balancing robots [41], has already been presented at an international conference. Furthermore, as a follow-up method, the method was developed by applying transfer learning and continual learning to expand the object handled by the proposed robot and is currently organizing data for publication.

## Figures and Tables

**Table 1:** Optimal policy convergence rate (OPCR) according to RGB image and grayscale image (α = 0.005, γ = 0.9).

Image Size	Optimal Policy Convergence Rate	
	RGB	Grayscale
128 × 170	90%	72%
84 × 112	86%	84%
84 × 84	84%	72%

**Table 2:** Optimal policy convergence rate (OPCR) of DQN according to random action ratio ε for optimal hyperparameter (α=0.005,γ=0.9).

Policy	Optimal Policy Convergence Rate
greedy	20%
10–greedy	90%
20–greedy	90%

**Table 3:** Comparison of the precision and the measured angle of the table for the proposed model (Figure 11) and baseline [39].

Model	Precision	State	Angle Variation	Angular Variation
Q-learning	67%	$s_{upup}$	8.2°⟹0.2°	−8.0°
with AlexNet [39]		$s_{down}$	−1.2°⟹0.30°	+1.5°
DQN (ours)	90%	$s_{upup}$	7.47°⟹0.66°	−6.81°
		$s_{down}$	−2.64°⟹−0.30°	+2.34°

## Data Availability

The Table-balancing dataset presented in this study is openly available in https://github.com/justcallkim/Table-balancing-Datasets.git github repository accessed on 31 May 2023.

## References

[1] Mnih V., Kavukcuoglu K., Silver D., Graves A., Antonoglou I., Wierstra D., Riedmiller M. (2013). Playing atari with deep reinforcement learning. arXiv.

[2] Watkins C.J., Dayan P. (1992). Q-learning. Mach. Learn..

[3] Schulman J., Levine S., Abbeel P., Jordan M., Moritz P. Trust region policy optimization. Proceedings of the International Conference on Machine Learning.

[4] Schulman J., Wolski F., Dhariwal P., Radford A., Klimov O. (2017). Proximal policy optimization algorithms. arXiv.

[5] Lillicrap T.P., Hunt J.J., Pritzel A., Heess N., Erez T., Tassa Y., Silver D., Wierstra D. Continuous control with deep reinforcement learning. Proceedings of the 4th International Conference on Learning Representations, ICLR 2016—Conference Track Proceedings.

[6] Merel J., Tassa Y., Srinivasan S., Lemmon J., Wang Z., Wayne G., Heess N. (2017). Learning human behaviors from motion capture by adversarial imitation. arXiv.

[7] Peng X.B., Berseth G., Van de Panne M. (2016). Terrain-adaptive locomotion skills using deep reinforcement learning. ACM Trans. Graph. (TOG).

[8] Lobos-Tsunekawa K., Leiva F., Ruiz-del Solar J. (2018). Visual navigation for biped humanoid robots using deep reinforcement learning. IEEE Robot. Autom. Lett..

[9] Wen S., Chen X., Ma C., Lam H.K., Hua S. (2015). The Q-learning obstacle avoidance algorithm based on EKF-SLAM for NAO autonomous walking under unknown environments. Robot. Auton. Syst..

[10] Silver D., Huang A., Maddison C.J., Guez A., Sifre L., Van Den Driessche G., Schrittwieser J., Antonoglou I., Panneershelvam V., Lanctot M. (2016). Mastering the game of Go with deep neural networks and tree search. Nature.

[11] Debnath S., Nassour J. Extending cortical-basal inspired reinforcement learning model with success-failure experience. Proceedings of the 4th International Conference on Development and Learning and on Epigenetic Robotics.

[12] Aşık O., Görer B., Akın H.L. (2018). End-to-end deep imitation learning: Robot soccer case study. arXiv.

[13] Danel M. (2017). Reinforcement learning for humanoid robot control. POSTER, May.

[14] Stulp F., Buchli J., Theodorou E., Schaal S. Reinforcement learning of full-body humanoid motor skills. Proceedings of the 2010 10th IEEE-RAS International Conference on Humanoid Robots.

[15] Levine S., Pastor P., Krizhevsky A., Ibarz J., Quillen D. (2018). Learning hand-eye coordination for robotic grasping with deep learning and large-scale data collection. Int. J. Robot. Res..

[16] Suay H.B., Chernova S. Effect of human guidance and state space size on interactive reinforcement learning. Proceedings of the 2011 Ro-Man.

[17] Wang C., Hindriks K.V., Babuska R. Active learning of affordances for robot use of household objects. Proceedings of the 2014 IEEE-RAS International Conference on Humanoid Robots.

[18] Kato Y., Kanda T., Ishiguro H. May I help you? Design of human-like polite approaching behavior. Proceedings of the 2015 10th ACM/IEEE International Conference on Human-Robot Interaction (HRI).

[19] Ozaki Y., Ishihara T., Matsumura N., Nunobiki T., Yamada T. Decision-making prediction for human-robot engagement between pedestrian and robot receptionist. Proceedings of the 2018 27th IEEE International Symposium on Robot and Human Interactive Communication (RO-MAN).

[20] Bergstrom N., Kanda T., Miyashita T., Ishiguro H., Hagita N. Modeling of natural human-robot encounters. Proceedings of the 2008 IEEE/RSJ International Conference on Intelligent Robots and Systems.

[21] Tuyen N.T.V., Jeong S., Chong N.Y. Emotional bodily expressions for culturally competent robots through long term human-robot interaction. Proceedings of the 2018 IEEE/RSJ International Conference on Intelligent Robots and Systems (IROS).

[22] Doering M., Glas D.F., Ishiguro H. (2019). Modeling interaction structure for robot imitation learning of human social behavior. IEEE Trans. Hum. Mach. Syst..

[23] Xue Y., Wang F., Tian H., Zhao M., Li J., Pan H., Dong Y. Proactive interaction framework for intelligent social receptionist robots. Proceedings of the 2021 IEEE International Conference on Robotics and Automation (ICRA).

[24] Yalçinkaya B., Couceiro M.S., Soares S.P., Valente A. (2023). Human-Aware Collaborative Robots in the Wild: Coping with Uncertainty in Activity Recognition. Sensors.

[25] Khan I.U., Afzal S., Lee J.W. (2022). Human activity recognition via hybrid deep learning based model. Sensors.

[26] Huang C.M., Mutlu B. Learning-based modeling of multimodal behaviors for humanlike robots. Proceedings of the 2014 9th ACM/IEEE International Conference on Human-Robot Interaction (HRI).

[27] Chi T.C., Shen M., Eric M., Kim S., Hakkani-tur D. Just ask: An interactive learning framework for vision and language navigation. Proceedings of the AAAI Conference on Artificial Intelligence.

[28] Thobbi A., Gu Y., Sheng W. Using human motion estimation for human-robot cooperative manipulation. Proceedings of the 2011 IEEE/RSJ International Conference on Intelligent Robots and Systems.

[29] Sheng W., Thobbi A., Gu Y. (2014). An integrated framework for human–robot collaborative manipulation. IEEE Trans. Cybern..

[30] Softbank NAO Humanoid Robot. https://www.softbankrobotics.com/.

[31] Qi W., Ovur S.E., Li Z., Marzullo A., Song R. (2021). Multi-sensor guided hand gesture recognition for a teleoperated robot using a recurrent neural network. IEEE Robot. Autom. Lett..

[32] Qi W., Aliverti A. (2019). A multimodal wearable system for continuous and real-time breathing pattern monitoring during daily activity. IEEE J. Biomed. Health Inform..

[33] Su H., Qi W., Schmirander Y., Ovur S.E., Cai S., Xiong X. (2022). A human activity-aware shared control solution for medical human–robot interaction. Assem. Autom..

[34] Schwung D., Csaplar F., Schwung A., Ding S.X. An application of reinforcement learning algorithms to industrial multi-robot stations for cooperative handling operation. Proceedings of the 2017 IEEE 15th International Conference on Industrial Informatics (INDIN).

[35] Rizk Y., Awad M., Tunstel E.W. (2019). Cooperative heterogeneous multi-robot systems: A survey. ACM Comput. Surv. (CSUR).

[36] Krüger J., Schreck G., Surdilovic D. (2011). Dual arm robot for flexible and cooperative assembly. CIRP Ann..

[37] Han R., Chen S., Hao Q. Cooperative multi-robot navigation in dynamic environment with deep reinforcement learning. Proceedings of the 2020 IEEE International Conference on Robotics and Automation (ICRA).

[38] The Vicon Motion Capture System. https://www.vicon.com/.

[39] Kim Y., Kang B.Y. (2020). Cooperative Robot for Table Balancing Using Q-learning. J. Korea Robot. Soc..

[40] Kim Y. (2020). Cooperative Robot Development Based on Deep Reinforcement Learning for Table Balancing. Master’s Thesis.

[41] Jeon H., Kim Y., Kang B. Interactive Reinforcement Learning for Table Balancing Robot. Proceedings of the the Joint Conference of the 59th Annual Meeting of the Association for Computational Linguistics (ACL-IJCNLP 2021) SpLU-RoboNLP Workshop.

